# Supplementary material for: Price convergence in grain markets with seasonal differences
Source: PLoS One. 2025 Dec 29;20(12):e0339577. doi: 10.1371/journal.pone.0339577 (PMC12758808; doi:10.1371/journal.pone.0339577)
Supplement: S1 Appendix — (PDF) [file pone.0339577.s001.pdf]

S1 Appendix. Robustness checks using FEWS NET price data

To assess whether the seasonal price patterns observed in our market survey are consistent with broader historical trends, we compare our results with Nigeria's staple food price data from the Famine Early Warning Systems Network (FEWS NET). Our dataset spans February 2022 to April 2023 and covers markets across a wide geographic range, while the FEWS NET series extends from October 2014 to April 2023 and includes 16 markets. Although our coverage is shorter, FEWS NET provides a valuable benchmark because of its long time series and established data collection methodology. For comparability, we match 5% broken rice in the FEWS NET data with local rice in our dataset, and milled rice in the FEWS NET data with long-grain rice in our dataset. Cowpea is directly comparable across both datasets. This matching allows us to examine whether the direction and shape of seasonal price variation in our single-year survey resemble the longer-term averages from FEWS NET.

When comparing the patterns, cowpea shows the clearest similarity (See Figure S1). In both datasets, prices are lower in the early post-harvest months and higher toward the end of the cycle, consistent with supply drawdown over time. In our data, the post-harvest decline is more pronounced, with a sharper trough around five to six months after harvest, whereas in FEWS NET the decline is more gradual, likely because the multi-year averaging smooths out sharp drops. The timing of the subsequent price increase is similar, beginning around the fifth or sixth month after harvest in both cases. For local rice and 5% broken rice, both datasets display relatively muted seasonality compared to cowpea, with prices remaining broadly stable for much of the year. In our data, prices drop slightly after harvest and then rise gradually, whereas in FEWS NET the trend is flatter, though the general stability is consistent. This lack of a pronounced post-harvest dip in either dataset aligns with the idea that rice's longer storage life and more integrated supply chains smooth seasonal fluctuations.

The comparison for long-grain rice and milled rice shows more divergence. In our dataset, prices peak during the harvest season, dip around months five to six, and then rise modestly toward the end of the cycle. In the FEWS NET data, by contrast, prices rise to a peak in the middle of the cycle and then decline toward the end. This mismatch may be due to differences in the underlying market composition, as our long-grain rice sample likely contains a higher share of imported rice subject to international price movements, whereas the FEWS NET milled rice series may reflect a mix of domestic and imported sources. It is also possible that the single year covered by our survey captures an atypical seasonal pattern not visible in the longer FEWS NET series.

Overall, the FEWS NET comparison offers partial corroboration of our results, particularly for cowpea and, to a lesser extent, local rice, where both datasets exhibit broadly similar post-harvest dynamics. However, it does not replicate our findings in all cases, and differences in product definitions, market coverage, and especially time span mean that the seasonal shapes are not always aligned. FEWS NET's multi-year averages tend to dampen the magnitude of seasonal swings, while our single-year data may capture sharper short-term fluctuations. These differences do not necessarily undermine the validity of our findings; rather, they highlight how market composition,

data scope, and the specific time period observed can influence the seasonal patterns captured in price data.

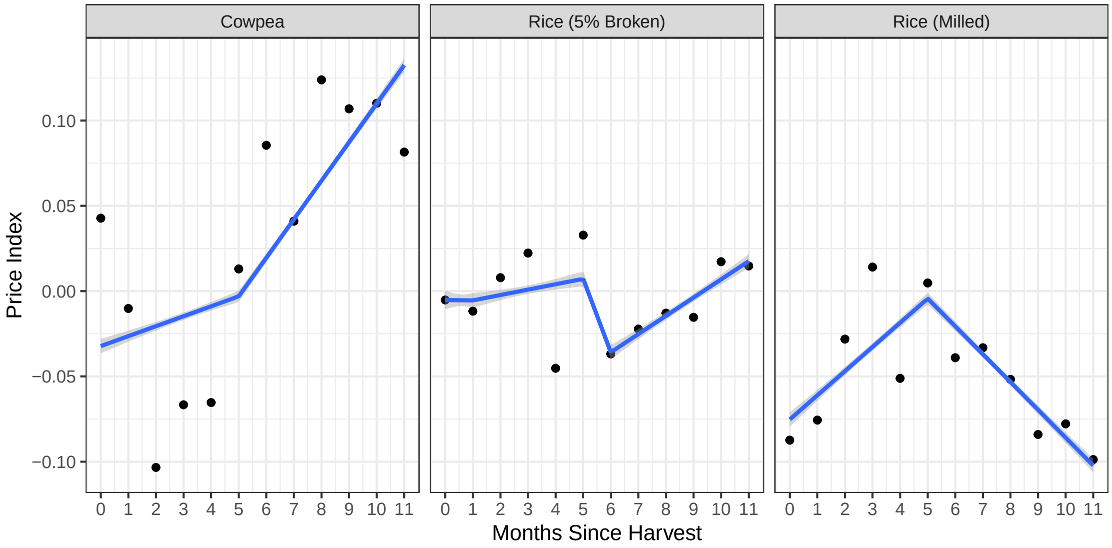

Figure S1: Seasonal price variation by item — FEWS NET data (Oct 2014 – Apr 2023)
