# Supplementary material for: Price convergence in grain markets with seasonal differences
Source: PLoS One. 2025 Dec 29;20(12):e0339577. doi: 10.1371/journal.pone.0339577 (PMC12758808; doi:10.1371/journal.pone.0339577)
Supplement: S2 Appendix — (PDF) [file pone.0339577.s002.pdf]

S2 Appendix. Transportation cost and spatial price variation

Table S2: Transportation cost and spatial price variation

	Dependent variable:					
	$\left\vert \log\left(\frac{P_{ikt}}{P_{jkt}}\right)\right\vert$					
		All products		Cowpea	Local Rice	Long-grain rice
	(1)	(2)	(3)	(4)	(5)	(6)
log(Distance)	0.064***		−0.110***	−0.092***	−0.290***	−0.128***
	(0.0004)		(0.003)	(0.004)	(0.009)	(0.007)
log(Time)		0.073***	0.191***	0.153***	0.441***	0.236***
		(0.0005)	(0.003)	(0.004)	(0.009)	(0.007)
log(Size of Origin)	0.002***	0.002***	0.002***	0.006***	0.006***	−0.005***
	(0.001)	(0.001)	(0.001)	(0.001)	(0.002)	(0.001)
log(Size of Destination)	0.009***	0.010***	0.013***	0.016***	0.026***	0.008***
	(0.0005)	(0.0005)	(0.0005)	(0.001)	(0.002)	(0.001)
Item fixed effect	Yes	Yes	Yes	No	No	No
Week fixed effect	Yes	Yes	Yes	Yes	Yes	Yes
Region fixed effect	Yes	Yes	Yes	Yes	Yes	Yes
Observations	311,288	311,288	311,288	140,808	55,322	49,340
R^2	0.201	0.207	0.211	0.198	0.315	0.266
Adjusted R^2	0.201	0.207	0.211	0.198	0.314	0.265
Note:						*p<0.1; **p<0.05; ***p<0.01

Table S2 presents regression estimates of the absolute log price ratio, $|\log(P_{ikt}/P_{jkt})|$, as a function of transportation cost variables and market size. Columns (1)–(3) report estimates for all products, while columns (4)–(6) focus on individual commodities—cowpea, local rice, and long-grain rice. When estimated for all products, a model including only log(Distance) (column 1) shows a positive and statistically significant association (0.064, $p < 0.01$), suggesting that greater distances are linked to larger price differences. However, in a model that includes both log(Distance) and log(Time) (column 3), the coefficient on log(Distance) turns negative (–0.110, $p < 0.01$) while log(Time) remains positively significant (0.191, $p < 0.01$), indicating that, holding travel time constant, an increase in distance is associated with a reduction in the price gap. Similar patterns emerge in the commodity-specific regressions, with local rice exhibiting the largest magnitude coefficients (–0.290 for log(Distance) and 0.441 for log(Time)), implying a stronger sensitivity of spatial price variation to travel time. In addition, the coefficients on log(Size of Origin) and log(Size of Destination) are positive and statistically significant across models, suggesting that larger market sizes contribute to greater price dispersion. The inclusion of item, week, and region fixed effects (and

the exclusion of item fixed effects in the commodity-specific models) helps control for unobserved heterogeneity, ensuring that the estimates capture the isolated impact of transportation costs on spatial price variation.
