# Supplementary material for: Price convergence in grain markets with seasonal differences
Source: PLoS One. 2025 Dec 29;20(12):e0339577. doi: 10.1371/journal.pone.0339577 (PMC12758808; doi:10.1371/journal.pone.0339577)
Supplement: S3 Appendix — (PDF) [file pone.0339577.s003.pdf]

S3 Appendix. Harvest timing by crop and region in Nigeria

Table S3: Harvest timing by crop and region in Nigeria

**Crops**	**Location**	**Start of harvest**	**End of harvest**
White cowpeas	South	June	September
White cowpeas	North	August	October
Brown cowpeas	South	June	September
Brown cowpeas	North	August	October
Rice	South	August	November
Rice	North	November	January
