# Supplementary material for: Price convergence in grain markets with seasonal differences
Source: PLoS One. 2025 Dec 29;20(12):e0339577. doi: 10.1371/journal.pone.0339577 (PMC12758808; doi:10.1371/journal.pone.0339577)
Supplement: S4 Appendix — (PDF) [file pone.0339577.s004.pdf]

S4 Appendix. Maddala–Wu unit-root test results for crop prices

Table S4: Maddala–Wu unit-root test results for crop prices

Crop type	Test statistic (Chi-sq)	Degrees of freedom	p-value
Local rice	4616.7	580	$< 2.2e-16$
Long-grain rice	5234.1	542	$< 2.2e-16$
Brown cowpea	5334.4	754	$< 2.2e-16$
White cowpea	4638.0	978	$< 2.2e-16$

We conducted the Maddala–Wu unit-root test, a panel-data unit-root test, to evaluate the stationarity of price differentials across various markets for different crops. Stationarity is crucial in time-series analysis, as it implies that the statistical properties of the series, such as mean, variance, and autocorrelation, remain constant over time, which is essential for reliable inference in models focusing on short-term adjustments [Hamilton, 1994].

The results, summarized in Table 1, strongly indicate stationarity for all crops analyzed. For local rice, the test statistic is 4616.7 with 580 degrees of freedom, resulting in a p-value of less than 2.2e-16. Similarly, long-grain rice, brown cowpea, and white cowpea exhibit test statistics of 5234.1 (df: 542), 5334.4 (df: 754), and 4638.0 (df: 978), respectively, all with p-values below 2.2e-16. These findings suggest that the price differentials are stationary, which justifies the use of short-term adjustment models in our subsequent analysis [Maddala and Wu, 1999].

References

James D. Hamilton. *Time Series Analysis.* Princeton University Press, Princeton, NJ, 1994.

G.S. Maddala and Shaowen Wu. A comparative study of unit root tests with panel data and a new simple test. *Oxford Bulletin of Economics and Statistics*, 61(S1):631–652, 1999.
