# Supplementary material for: Price convergence in grain markets with seasonal differences
Source: PLoS One. 2025 Dec 29;20(12):e0339577. doi: 10.1371/journal.pone.0339577 (PMC12758808; doi:10.1371/journal.pone.0339577)
Supplement: Attachment — Submitted filename: PlosOneRound2Revs1.docx [file pone.0339577.s007.docx]

PONE-D-25-30518R1

Price Convergence in Grain Markets with Seasonal Differences

PLOS ONE

**Response to Editor.**

Thanks for sharing this feedback from the reviewers. We are thankful that Reviewer 2 found the revisions we made acceptable. We have addressed the additional requests from Reviewer 1 in the clearest way that we think will address the stated concerns.

As directed, we have included with this revision, a rebuttal/response document, (that is, this document), a clean updated manuscript with suggested revisions, and a marked-up manuscript that shows the changes made.

We look forward to the next step, and hopefully, hearing that the revisions satisfied the Reviewer 1’s request for minor changes.

Sincerely,

M. Olabisi.

Michigan State University.

**Reviewer 1:**

*Thank you for addressing my comments. I have some last minor points (numbers refer to my numbered comments on the previous version):*

**Reviewer Comment** 0:

*The new section on the Theoretical Framework is helpful. However, there are two points of confusion. First, line 62-63: This is not quite right. It is equation 1 that delivers the seasonal pattern by itself (prices are higher in non-harvest months because supply is coming from storage from the previous harvest). Equation 2 is just a reduced-form representation of the implications of equation 1. Equation 2 would make more sense if you present this more clearly as a 2-period model (harvest vs. non-harvest) rather than monthly. Prices are higher in the non-harvest period because of storage costs. Second, line 81-83: It was not clear what you meant by “domestic harvest enters weakly” as the domestic harvest does not appear in equation 5. Either include it explicitly in equation 5 or don’t mention it in the sentence. Also, why does equation 5 hold approximately and not with equality? It might also help to include another equation here after equation 5 that would be analogous to equation 4 but for the imported variety case.*

**Response to Reviewer Comment** 0:

We are thankful for this hint from the reviewer. We have now revised the theoretical framework accordingly.

First, we now make it explicit that the storage no-arbitrage condition alone generates the seasonal pattern. To do so, we introduce a simple two-period example (harvest vs. non-harvest) and explain that prices are higher in the non-harvest period because supply comes from stored stocks and must cover storage costs.

Second, in the subsection on imported varieties, we removed the phrase “domestic harvest enters weakly” since harvest does not appear in that equation, and we now write the imported price relationship with equality rather than approximately. As you suggested, we also add a brief expression for the cross-market price gap for imports, directly analogous to the domestic-gap case. These changes address the points of confusion you raised. The updates can be found in line 64-76 and line 92-99.

**Reviewer Comment** 1:

*The map is helpful but leaves me wondering two things. Why do some markets appear to have no links? And why are there no markets in Lagos and vicinity? It would be helpful to clarify in the text. Perhaps add more about the snowball approach or how the starting markets were selected.*

**Response to Reviewer Comment** 1:

We are thankful for the guidance from the reviewer. We should start by admitting that Lagos was excluded largely because our limited budget could not cover it. Our snowball approach started from the largest known grain markets in the key production areas – predominantly in Northern Nigeria. These include Dawanau near Kano and Mutum Biu in Taraba State. Given our extensive coverage of Bodija market in Ibadan (about 70 miles from Lagos), and past research that shows the significance of Bodija to price movements in the southwest of Nigeria, we focused on the 97 markets used for this paper, with a reasonable level of confidence that our main findings, if we had the budget to include Lagos, would not be significantly different from our main findings as they are.

We are thankful that the reviewer used the phrase “appear to” in the question. We selected key link-markets that connect farms to consumers for our price surveys, but a few of these were not linked to other key markets – largely being linked to local food grain markets that are more than two steps removed from other key grain markets. Given that we could not track prices in all markets that we visited in 2022 - roughly 300 of them, there was no reason to plot a network graph that includes markets for which we have no price data. Furthermore, a network graph with 97 nodes is more legible and instructive than one with three times that number. In sum, the network graph reflects only linkages between key markets, specifically, the 97 markets for which we track prices.

We have now updated the paper to explain that we exclude Lagos because of budget limits, and why a few nodes in the graph appear to have no connections, because they had no reported links to other key grain markets that we track in our data.

The updates can be found near line 177-190 of the revised paper.
